# Sex disparities in body composition trends among elementary school students based on grade

**DOI:** 10.3389/fped.2025.1600758

**Published:** 2026-06-17

**Authors:** Stefan Đorđević, Bojan Jorgić, Saša Milenković, Miljan Hadžović, Mima Stanković, Stănescu Rareș Cristian, Coja Daniel Mădălin, Ștefănescu Cătălin Aurelian, Ilie Onu, Daniel-Andrei Iordan

**Affiliations:** 1 Faculty of Sport and Physical Education, University of Niš, Niš, Serbia; 2 Faculty of Physical Education and Sport, National University of Physical Education and Sport, Bucharest, Romania; 3 Research Centre for Physical Therapy and Rehabilitation, “Dunărea de Jos” University of Galati, Galati, Romania; 4 Faculty of Physical Education and Sport, Dunarea de Jos University, Galați, Romania; 5 Faculty of Medical BioEngineering, Grigore T. Popa University of Medicine and Pharmacy, Iași, Romania

**Keywords:** children, detection, BMI, anthropometrics, body composition

## Abstract

**Introduction:**

Modern lifestyle changes have contributed to developments and advances in almost all spheres of a child's life. However, negative consequences such as hypokinesia and obesity are also present. This study aimed to examine the differences in body composition parameters between boys and girls in higher elementary school grades. The sample consisted of 484 school-aged boys and girls from grades V to VIII in Knjaževac. The municipality of Knjaževac represents a group of municipalities with up to 30,000 inhabitants, a municipality of that size based on the number of inhabitants is 49.6% of the total number of municipalities in the Republic of Serbia.

**Methods:**

An anthropometer (SECA model 284; SECA, Hamburg, Germany) and a body composition analyzer (InBody 770, InBody Co, Seoul, Korea) were used to collect the measured parameters.

**Results:**

Using ANOVA, with the addition of post hoc analysis, a statistically significant trend was determined across all analyzed body composition parameters, namely, skeletal muscle mass (SMM), body fat mass, lean body mass (LBM), and relative lean body mass (RLBM) (*p* < 0.001, *p* = 0.049, *p* < 0.001, and *p* < 0.001, respectively), except for relative body fat mass (RBFM) (*p* = 0.145), in children of both sexes from grades V to VIII. A significant trend was observed among the boys for SMM, RSMM, LBM, RLBM (*p* < 0.001), and RBFM (*p* = 0.004), while among the girls, a significant trend was noted for SMM and LBM *p* < 0.001).

**Discussion:**

Sex-related differences in muscle mass and body mass were clearly observed. In the male subjects, there was a significant increase in relative muscle mass, while in the female subjects, there was a statistically significant increase in body fat percentage. These findings highlight the necessity of taking sex differences into account when monitoring and analyzing body composition parameters in children.

## Introduction

The modern era has brought numerous improvements that enhance daily life, but also negative factors that diminish its quality, particularly those affecting health and development. Health is a critical factor during periods of rapid growth and development (1). Body composition, as one of the parameters of health, is an important factor in the phases of intensive growth and development of an organism, and is especially important in the period of early adolescence (2). Increasing body fat and body mass index (BMI) values are becoming increasingly common and negatively impact children's health and development (3). The global prevalence of obesity among children and adolescents has increased significantly in recent decades, with estimates that by 2030, almost 30% of children will be obese, while in middle- and high-income countries, this percentage will reach 58.3%. According to recent studies, the incidence of overweight and obesity among children and adolescents in the Balkan region ranges from 25% to 40%, with differences based on country, age group, and urban vs. rural settings. This emphasizes a major public health risk that necessitates focused responses.

During the entire period of growth and development, there are noticeable differences between boys and girls. Differences also exist in newborns, where girls exhibit significantly higher relative fat mass than boys (4). Nevertheless, by 6 months of age, no sexual dimorphism in body composition is observed (4).

Nutritional status, as suggested by Cavazzotto et al. (5), was determined mainly by body weight and height alone, using BMI. Today, however, assessments are more often based on analyses of body composition and fat distribution as they can provide a more realistic picture (6, 7). Furthermore, body composition can be measured more precisely with modern diagnostic equipment, which can help determine the differences more clearly (6).

Patton et al. (8) found that during the early adolescent years (10–15), key physical development traits encompass rapid growth. This phase, as stated by Norris et al. (9), is marked by notable increases in height, weight, and the development of skeletal and muscular systems. Čech et al. (10) conducted research in which they divided the boys and girls into three groups: group 1 (11.4 ± 0.4 years old), group 2 (13.0 ± 0.6), and group 3 (14.8 ± 0.5). They observed a significant year-to-year increase in fat-free mass and skeletal muscle mass (SMM) for the boys between all three groups (*p* < 0.05). In the girl groups, differences were noted in fat-free mass and skeletal muscle mass, but only between the first and second group, with no differences in relation to the third group, and no differences in body fat percentage. These findings are in accordance with Durá-Travé et al. (11), who stated that fat-free mass increases with age. Moreover, Bitar et al. (12) proved that at the onset of puberty, both boys and girls experienced an increase in fat-free mass of 4.7 ± 2.1 kg per year. In contrast, the gain in fat mass was 1.0 ± 1.2 kg per year in girls and 0.20 ± 0.66 kg per year in boys.

Although large-scale studies on adolescent populations exist (13), and some include more anthropometric variables (14), no research to date has focused specifically on differences across school grades and sexes. To the best of the authors' knowledge, no prior study has examined this wide a range of anthropometric variables across multiple grade levels for each sex separately. Furthermore, the point of differentiation remains unclear. Therefore, the aim of this research was to determine the differences in body composition parameters between boys and girls in different elementary school grades.

## Materials and methods

### Participants

The sample consisted of school-age children from the fifth to the eighth grades (Vn = 152; VIn = 114; VIIn = 117; and VIIIn = 101). The subjects were students from all primary schools in the municipality of Knjaževac, Republic of Serbia. A detailed description of the subjects is shown in Table 1. All the measurements included in this research were carried out according to the Declaration of Helsinki from 2008 (World Medical Association, 2011) (15). The study was approved by the Ethics Committee of the University of Niš under no. 04-542.

**Table 1:** Participant characteristics.

Grade	Sex	Body height (mean ± SD)	Body mass (mean ± SD)	BMI (mean ± SD)
	T (484)	154.96 ± 10.11	50.36 ± 14.50	20.69 ± 4.54
	M (252)	155.90 ± 11.14	51.43 ± 15.96	20.84 ± 4.82
	F (232)	153.95 ± 8.75	49.19 ± 12.65	20.54 ± 4.22
V	M (78)	147.26 ± 6.88	43.33 ± 13.08	19.77 ± 4.94
	F (74)	147.29 ± 8.12	43.69 ± 12.25	19.90 ± 4.33
	T (152)	147.28 ± 7.49	43.51 ± 12.64	19.83 ± 4.64
VI	M (55)	153.52 ± 9.05	49.17 ± 13.05	20.68 ± 4.18
	F (59)	153.81 ± 6.75	48.54 ± 13.31	20.32 ± 4.55
	T (114)	153.67 ± 7.91	48.84 ± 13.13	20.49 ± 4.36
VII	M (61)	159.98 ± 10.13	56.76 ± 17.11	21.86 ± 4.86
	F (56)	153.67 ± 6.73	53.65 ± 11.84	21.42 ± 4.19
	T (117)	158.88 ± 8.71	55.27 ± 14.84	21.65 ± 4.54
VIII	M (58)	165.48 ± 8.87	58.86 ± 15.48	21.35 ± 4.98
	F (43)	160.74 ± 6.37	53.77 ± 9.47	20.79 ± 3.42
	T (101)	163.46 ± 8.21	56.69 ± 13.44	21.11 ± 4.38

M, male; F, female; T, total.

## Procedure

### Anthropometric parameters

A standardized state-of-the-art anthropometric anthropometer (SECA model 284; SECA, Hamburg, Germany) was used to assess the anthropometric parameters of the subjects, which complies with the highest criteria established by the International Society for the Advancement of Kinanthropometry (16).

### Body composition parameters

An InBody 770 (InBody Co., Seoul, Korea) was used to assess the body composition parameters of the subjects. The reliability of this instrument, i.e., validity and reliability, has been determined by previous research (17–19). The research used recommendations for performing and reporting bioelectrical impedance analysis (BIA) measurements based on the results and conclusions of a systematic review (20). This instrument performs a five-segment analysis of body composition and uses frequencies of 1, 5, 50, 250, and 500 kHz for this assessment.

The protocol required that the subjects included in this testing had no chronic diseases, that they had not consumed food or drink for at least 8 h, and that they had emptied their bladders on the morning before the test, which was organized between 9:00 and 12:00 a.m. in the laboratories of the diagnostic center within the Sports Association of Knjaževac.

Testing guidelines from (21) were followed during the testing process. The measurement procedure required that, before the diagnosis itself, the subject had to be dressed only in underwear and have no metal objects on or inside them. After this, the subject climbed onto a platform, where they were in an upright position with their feet in parallel position on the intended fields for their toes and heels and their arms were slightly away from the body with sticks in their hands. After the correct placement of the body segments by following the steps presented on the instrument display, the analysis process lasted 50s. The diagnostic results were then read on the integrated computer. To evaluate body composition, the following variables were used: SMM expressed in kilograms, relative muscle mass (RMM) expressed as a percentage of total body mass, body fat mass (BFM) expressed in kilograms, relative body fat mass (RBFM) expressed as a percentage of total body mass, lean body mass (LBM) expressed in kilograms, and relative lean body mass (RLBM) expressed as a percentage of total body mass, Table 2. RMM and RLBM were calculated using the following formulae:

$$\begin{array}{c} \mathrm{RMM}=\left(\frac{\mathrm{Skeletal}\;\mathrm{Muscle}\;\mathrm{Mass}(\mathrm{SMM})}{\mathrm{Total}\;\mathrm{Body}\;\mathrm{Mass}}\right)\;\times 100,\\ \mathrm{RLBM}=\left(\frac{\mathrm{Lean}\;\mathrm{Body}\;\mathrm{Mass}(\mathrm{LBM})}{\mathrm{Total}\;\mathrm{Body}\;\mathrm{Mass}}\right)\;\times 100 \end{array}$$

**Table 2:** Results of the statistical analyses of body composition parameters.

Gr	Sex	*n*	SMM (kg)	RMM (%)	BFM (kg)	RBFM (%)	LBM (kg)	RLBM (%)
T	M	252	20.96 ± 5.61	41.56 ± 5.56	12.58 ± 9.51	22.39 ± 10.52	32.12 ± 9.01	63.39 ± 8.35
	F	232	18.65 ± 3.72	38.64 ± 4.54	14.07 ± 7.99	26.87 ± 9.07	28.53 ± 6.18	58.79 ± 6.08
	T	484	19.85 ± 4.93	40.16 ± 5.30	13.30 ± 8.84	24.54 ± 10.09	30.40 ± 7.98	61.19 ± 7.69
V	M	78	16.93 ± 3.09	40.38 ± 5.46	11.22 ± 9.06	23.03 ± 11.07	25.69 ± 5.38	60.79 ± 7.07
	F	74	16.25 ± 3.40	38.11 ± 4.96	12.63 ± 7.97	26.73 ± 10.11	24.60 ± 5.59	57.33 ± 6.34
	T	152	16.59 ± 3.24	39.27 ± 5.33	11.91 ± 8.54	24.82 ± 10.74	25.16 ± 5.49	59.11 ± 6.92
VI	M	55	19.29 ± 4.03	40.10 ± 4.84	13.21 ± 8.10	24.70 ± 9.86	29.50 ± 6.57	61.03 ± 6.65
	F	59	18.72 ± 3.59	39.46 ± 4.66	13.33 ± 8.52	25.45 ± 9.29	28.53 ± 5.93	59.85 ± 6.13
	T	114	18.99 ± 3.80	39.76 ± 4.73	13.27 ± 8.28	25.08 ± 9.53	29.00 ± 6.24	60.42 ± 6.39
VII	M	61	22.99 ± 5.65	41.34 ± 5.35	14.46 ± 9.85	23.55 ± 9.97	35.26 ± 8.83	63.34 ± 8.48
	F	56	20.42 ± 3.20	38.72 ± 4.17	15.45 ± 7.86	27.37 ± 8.43	31.36 ± 5.44	59.23 ± 5.85
	T	117	21.75 ± 4.79	40.08 ± 4.97	14.93 ± 8.92	25.37 ± 9.42	33.39 ± 7.63	61.37 ± 7.60
VIII	M	58	25.82 ± 4.83	44.78 ± 5.37	11.83 ± 10.76	18.13 ± 9.95	39.95 ± 7.24	69.19 ± 8.55
	F	43	20.37 ± 2.75	38.31 ± 4.05	15.77 ± 7.04	28.40 ± 7.54	31.61 ± 4.60	59.29 ± 5.55
	T	101	23.49 ± 4.88	42.02 ± 5.80	13.50 ± 9.52	22.49 ± 10.31	36.40 ± 7.75	64.98 ± 8.88

Gr, grade; M, male; F, female; T, total; SMM, skeletal muscle mass; RММ, relative muscle mass; BFM, body fat mass; RBFM, relative body fat mass; LBM, lean body mass; RLBM, relative lean body mass.

### Procedure

The data obtained from the measurements were processed using statistical packages within SPSS 20 (Statistical Package for Social Sciences, v20.0, SPSS Inc., Chicago, IL, USA). Descriptive statistics parameters, i.e., central and dispersion parameters, were calculated for all the variables used to determine body composition and are presented as arithmetic mean (AS) and standard deviation (SD). One-factor analysis of variance (ANOVA), with the addition of least significant difference (LSD) if equal variance was assumed and Tamhane’s T2 test if equal variance was not assumed, was used to determine the trend in changes in body composition parameters in children of different sexes and grades in elementary school. Analysis of covariance (ANCOVA) was used to estimate the influence of covariates (body height, body mass, and BMI) on changes in body composition parameters. Eta squared was used to determine the effect size. In all the statistical analyses, significance was considered when *p* < 0.05.

## Results

### Male participants

Significant differences between the grades were observed (*p* < 0.001), with only the difference between the seventh and eighth grades not being significant (*p* = 0.052). Furthermore, the results showed statistically significant differences in both relative muscle mass and lean body mass (*p* < 0.001). Based on the results, a significant difference between the grades was observed in both skeletal muscle mass and lean body mass (*p* < 0.001). In contrast, absolute body fat mass showed no statistically significant difference between male subjects in different grades (*p* = 0.206), while *the post hoc* analysis determined that there was only a statistically significant difference between subjects in the fifth and seventh grades (*p* = 0.047). The relative body fat mass results showed statistically significant differences (*p* = 0.004), while the *post hoc* analysis determined a high-level statistically significant difference in this variable between subjects in fifth and eighth grades (*p* = 0.007), sixth and eighth grades (*p* < 0.001), and seventh and eighth grades (*p* = 0.004).

### Female participants

The absolute skeletal muscle mass results showed a statistically significant difference between the grades (*p* < 0.001); however, there was no difference between the seventh and eighth grades (*p* = 0.940). Furthermore, the results showed no difference in relative muscle mass between the grades (*p* = 0.365). Moreover, there was no statistically significant difference in absolute body fat mass (*p* = 0.088) between female subjects in different elementary school grades. Relative body fat mass showed no statistically significant difference (*p* = 0.416). Lean body mass showed a statistically significant difference between the grades (*p* < 0.001), except for between the seventh and eighth grades (*p* = 0.820). The relative lean body mass results did not show a statistically significant difference between different grades (*p* = .084), except for between the fifth and sixth grades (*p* = 0.017).

## Discussion

The results generally indicated a statistically significant trend in changes in certain parameters of body composition in the total sample (Table 3) and by sex and grades (Tables 4, 5).

**Table 3:** Trend in changes in body composition parameters in children of both sexes in older grades in elementary school.

Gr	SMM (kg)				RMM (%)				BFM (kg)				RBFM (%)				LBM (kg)				RLBM (%)			
	V	VI	VII	VIII	V	VI	VII	VIII	V	VI	VII	VIII	V	VI	VII	VIII	V	VI	VII	VIII	V	VI	VII	VIII
V		0.000	0.000	0.000		0.444	0.208	0.000		0.212	0.005	0.157		0.837	0.657	0.072		0.000	0.000	0.000		0.155	0.013	0.000
VI	0.000		0.000	0.000	0.444		0.647	0.002	0.212		0.151	0.844	0.837		0.825	0.061	0.000		0.000	0.000	0.155		0.329	0.000
VII	0.000	0.000		0.052	0.208	0.647		0.006	0.005	0.151		0.233	0.657	0.825		0.036	0.000	0.000		0.001	0.013	0.329		0.000
VIII	0.000	0.000	0.052		0.000	0.002	0.006		0.157	0.844	0.233		0.072	0.061	0.036		0.000	0.000	0.001		0.000	0.000	0.000	
HT (LSD; Tam)(sig)	0.000				0.375				0.750				0.345				0.000				0.007			
p	0.000				0.001				0.049				0.145				0.000				0.000			
P + cov	0.048				0.070				0.051				0.120				0.017				0.025			
EK	0.294				0.036				0.016				0.011				0.128				0.021			

Gr, grade; HT, homogeneity test; LSD, least significant difference; Tam, Tamhane’s test; SMM, skeletal muscle mass; RMM, relative muscle mass; BFM, body fat mass; RBFM, relative body fat mass; LBM, lean body mass; RLBM, relative lean body mass; ЕК, еta coefficient; P + cov, significant difference between scores after taking covariates into account (body height, body mass, and BMI).

**Table 4:** Trend in changes in body composition parameters in boys in older grades in elementary school.

Gr	SMM (kg)				RMM (%)				BFM (kg)				RBFM (%)				LBM (kg)				RLBM (%)			
	V	VI	VII	VIII	V	VI	VII	VIII	V	VI	VII	VIII	V	VI	VII	VIII	V	VI	VII	VIII	V	VI	VII	VIII
V		0.003	0.000	0.000		0.763	0.288	0.000		0.235	0.047	0.712		0.358	0.766	0.007		0.003	0.000	0.000		0.863	0.054	0.000
VI	0.003		0.000	0.000	0.763		0.207	0.000	0.235		0.480	0.439	0.358		0.550	0.001	0.003		0.000	0.000	0.863		0.108	0.000
VII	0.000	0.000		0.024	0.288	0.207		0.000	0.047	0.480		0.132	0.766	0.550		0.004	0.000	0.000		0.000	0.054	0.108		0.000
VIII	0.000	0.000	0.024		0.000	0.000	0.000		0.712	0.439	0.132		0.007	0.001	0.004		0.000	0.000	0.000		0.000	0.000	0.000	
TH (LSD; Tam)(sig)	0.000				0.809				0.838				0.294				0.000				0.317			
p	0.000				0.000				0.206				0.004				0.000				0.000			
P + cov	0.000				0.000				0.000				0.000				0.000				0.000			
EK	0.384				0.107				0.018				0.052				0.168				0.030			

Gr, grade; HT, homogeneity test; LSD, least significant difference; Tam, Tamhane’s test; SMM, skeletal muscle mass; RMM, relative muscle mass; BFM, body fat mass; RPBF, relative body fat mass; LBM, lean body mass; RLBM, relative lean body mass; ЕК, еta coefficient; P + cov, Significant difference between scores after taking covariates into account (body height, body mass, and BMI).

**Table 5:** Trend in changes in body composition parameters in the girls in older grades in elementary school.

Gr	SMM (kg)				RMM (%)				BFM (kg)				RBFM (%)					LBM (kg)				RLBM (%)			
	V	VI	VII	VIII	V	VI	VII	VIII	V	VI	VII	VIII	V		VI	VII	VIII	V	VI	VII	VIII	V	VI	VII	VIII
V		0.000	0.000	0.000		0.089	0.453	0.824		0.617	0.046	0.040			0.972	0.999	0.894		0.000	0.000	0.000		0.017	0.077	0.093
VI	0.000		0.006	0.013	0.089		0.378	0.204	0.617		0.152	0.125	0.972			0.818	0.394	0.000		0.006	0.006	0.017		0.582	0.640
VII	0.000	0.006		0.940	0.453	0.378		0.656	0.046	0.152		0.841	0.999		0.818		0.988	0.000	0.006		0.820	0.077	0.582		0.964
VIII	0.000	0.013	0.940		0.824	0.204	0.656		0.040	0.125	0.841		0.894		0.394	0.988		0.000	0.006	0.820		0.093	0.640	0.964	
TH (LSD; Tam) (sig)	0.533				0.074				0.333					0.020				0.657				0.260			
p	0.000				0.365				0.088					0.416				0.000				0.084			
P + cov	0.009				0.002				0.004					0.001				0.055				0.010			
K	0.228				0.013				0.028					0.012				0.091				0.020			

Gr, grade; HT, homogeneity test; LSD, least significant difference; Tam, Tamhane’s test; SMM, skeletal muscle mass; RMM, relative muscle mass; BFM, body fat mass; RPBF, relative body fat mass; LBM, lean body mass; RLBM, relative lean body mass; ЕК, еta coefficient; P + cov, significant difference between scores after taking covariates into account (body height, body mass, and BMI).

The absolute skeletal muscle mass results found a statistically significant difference between the grades (*p* < 0.001). In accordance with this, previous research found a constant increase in muscle mass in children aged 10–15 years (22). Furthermore, as shown in Table 3, it can be seen that on the whole, a statistically significant difference between the grades was observed for the relative muscle mass variable (*p* < 0.001). Furthermore, the analysis of the influence of covariates was significant (*p* = 0.070). A more detailed analysis indicated that the greatest changes in this parameter occurred between the seventh and eighth grades. Although a statistically significant difference in SMM was found between the seventh and eighth grades, it is crucial to note that such a numerical difference does not always imply clinically or physiologically meaningful changes. Changes in body composition during adolescence are heavily impacted by pubertal maturation, which includes hormonal changes and increased development. As a result, increases in muscle mass may represent both normal growth and individual variances in pubertal development. Future research should incorporate an assessment of pubertal stage or hormonal indicators to better distinguish the effects of growth and maturation.

Similarly, the results indicated a statistically significant trend in changes in absolute body fat mass in children in higher grades in elementary school (*p* = 0.049), with the greatest difference observed between the fifth and seventh grades (*p* = 0.005). This indicates that the seventh grade is the time period when the peak occurs and that after this period, it decreases, while absolute and relative skeletal muscle mass and lean body mass have statistically significant growth precisely between the seventh and eighth grades. The relative body fat mass results indicated a general absence of a statistically significant trend (*p* = 0.145). In addition, the results of previous research show that there is also an increase in body fat mass in kilograms at this age (23). The results and these variables confirm that the period of the seventh and eighth grades is a period of more intensive changes in body composition.

The absolute lean body mass results indicated a statistically significant trend (*p* < 0.001). In a more detailed analysis, a statistically significant difference was observed between all the grades (*p* < 0.001). Previous research has also shown an increase in this variable in children from 10 to 15 years of age; however, in these studies, the analyzed sample was aged 5 to 20 years (24). The results of these studies showed that there are sex differences and that statistically significant growth is clearly observed in male subjects, while in female subjects, this growth is most intense up to the age of 12, and after this, the growth is insignificant. Furthermore, an increase in absolute lean body mass can be influenced by genetic and environmental factors (25, 26) and the level of secretion of growth hormones, i.e., androgens and testosterone (27). Lower absolute lean body mass values in children can have consequences in later life, such as a decrease in functional abilities, osteoporosis, and a low level of physical ability (28).

The relative lean body mass results showed a statistically significant trend (*p* < 0.001). In previous research, statistically significant changes in this parameter were also observed (24). Furthermore, absolute lean body mass has a high level of genetic predisposition, and its growth highly correlates with body mass, muscle mass, mineral levels, and estrogen and testosterone secretion. Taking into account the influence of covariates (weight, height, and BMI), there was still a statistically significant change in the following body composition parameters: SMM, BFM, LBM, and RLBM.

As shown by the skeletal muscle mass results in Table 4, a statistically significant difference was observed between the grades (*p* < 0.001) in both male and female subjects. However, the *post hoc* analysis found no statistical significance between seventh and eighth grades among the female subjects (*p* = 0.940). Previous research has confirmed these trends (22, 29). An explanation for this trend can be found in the general increase in total body mass, which is more pronounced in male subjects, but also in the intensive biological development in this age period that affects sexual maturation (28). The male subjects recorded a significant increase in the mean values of this parameter in the seventh and eighth grades, while in female subjects, it significantly increased between the sixth and seventh grades. Previous research (22, 29) also indicated an increase in absolute muscle mass in both sexes. Chiplonkar et al. (23) confirmed the time period of a significant increase in muscle mass as well as sex differences, which can be explained by sex differences in this age period (30) and by both an increase in the total body mass and by participating in more organized physical activities among male subjects (29).

The results in Tables 4, 5 show a statistically significant difference between the grades (*p* < 0.001) in male subjects, while there was no difference in female subjects (*p* = 0.365). This analysis established the existence of statistically significant differences (*p* < 0.001) between the fifth and eighth, sixth and eighth, and seventh and eighth grades in the male subjects, while no statistically significant difference between grades was found in the female subjects. Thus, we observed a constant increase, but also a significantly higher increase in the male subjects between the seventh and eighth grades, while it was almost unchanged in the female subjects. By comparing the obtained results with previous research (23), a greater increase in this parameter in male subjects, as well as a period of sudden increase, is clearly confirmed. Furthermore, it is indicated that the relative muscle mass of male subjects is higher compared to female subjects, and that the period in which the differences emerge is a period of intense biological maturation, i.e., a period of early puberty (30).

Regarding body fat percentage, a statistically significant difference between the grades was observed (*p* = 0.004) among the male subjects, while this was not the case for the female subjects (*p* = 0.416). Under the influence of covariance, statistically significant differences were also observed at the general level and among the girls (*p* < 0.001). Statistically significant differences were observed between the fifth and eighth grades, sixth and eighth grades, and seventh and eighth grades (*p* = 0.007, 0.001, and 0.004, respectively), while no statistically significant differences were found among the female subjects. The results were confirmed by previous research (23).

The absolute fat-free body mass results showed a statistically significant trend in subjects of both sexes (M, *p* < 0.001; Z, *p* = 0.055). Based on these results, the biggest difference between the subjects of each sex was present in the eighth grade. These findings are in accordance with the results of a previous study on a sample of the same ages, where a difference in the trend and mean values of male and female subjects was also observed in favor of the male subjects (24). This can be explained by changing body mass, body height, fat, and muscle mass values, as well as intense biological changes, in the body (27).

The relative fat-free body mass results indicated that there was a statistically significant difference between the grades (*p* < 0.001) among the male subjects, while this was not observed among the female subjects (*p* = 0.084). Under the influence of covariance, statistically significant differences were also observed at the general level and among the girls (*p* < 0.010). The *post hoc* analysis established the existence of statistically significant differences (*p* < 0.001) between the fifth and eighth, sixth and eighth, and seventh and eighth grades in the male subjects, while in the female subjects, no statistically significant differences were determined between the grades, except for between the fifth and sixth grades (*p* = 0.017). In accordance with this, previous research has also shown a statistically significant trend in changes in this parameter as well as a sex difference (24). The rationale for this can be found in comparative changes in relative muscle mass and body fat percentage. That is, if the body fat percentage and total body mass are increased and muscle mass is decreased, the lean body mass percentage must be lower, which is the case with female subjects, while this is completely the opposite in male subjects. The period of increased changes in muscle mass percentage, fat percentage, and lean body mass is different between the sexes, as the period of intense changes in these variables begins earlier in female subjects and the greatest growth occurs around the age of 12.

The results of this study show that a sex difference was present in the older grades (through the analysis of four grades). The difference can be explained, as previously assessed, by the physiognomy of the male and female bodies, but also by the unequal physical activity that was present in this study and between the sexes, as presented in previous research (31). Furthermore, studies have established that increased fat contributes to many health problems (e.g., cardiorespiratory issues, diabetes, and kidney diseases), while the opposite is true when it comes to increased muscle mass and lean body mass (32–34).

## Conclusions

Early detection and monitoring of body composition parameters in childhood can be the first step toward monitoring and eliminating chronic obesity and the possible consequences of obesity as a modern disease. The results of our research show that there are differences in body composition parameters between older school-age boys and girls. A shortcoming of this study is that these results may have been influenced by the maturation and growth of the children in this period, so we suggest that in further research, the goal should be to monitor body composition and the children’s biological and chronological maturity. Furthermore, one of the recommendations for further research is the correlation of body composition and the material and social status of children. The contribution of this study is reflected in the collection and analysis of the body composition parameters of children from Eastern Serbia and a recognition of the possible consequences of obesity.

## Data Availability

The datasets presented in this article are not readily available because they contain participants' information. Requests to access the datasets should be directed to the first author, Stefan Đorđević, dcre@unefs.ro.
